# Faith leaders’ messaging is essential to enhance HIV prevention among black Americans: results from the 2016 National Survey on HIV in the black community (NSHBC).

**DOI:** 10.1186/s12889-018-6301-0

**Published:** 2018-12-19

**Authors:** Yusuf Ransome, Laura M. Bogart, Amy S. Nunn, Kenneth H. Mayer, Keron R. Sadler, Bisola O. Ojikutu

**Affiliations:** 1 Department of Social and Behavioral Sciences, Yale School of Public Health, 60 College Street, LEPH, New Haven, CT 06510 USA; 2 RAND Corporation, Santa Monica, CA USA; 3 Brown School of Public Health, Providence, RI USA; 4 Harvard Medical School and Beth Israel Deaconess Medical Center, and Fenway Health, Boston, MA USA; 5 National Association for the Advancement of Colored People (NAACP), Baltimore, MD USA; 6 Brigham and Women’s Hospital and Harvard Medical School, Boston, MA USA

**Keywords:** HIV prevention; Black American; African-American; Black Church; PrEP; pre-exposure prophylaxis; Religion; Faith Leaders

## Abstract

**Background:**

To investigate whether religious service attendance and faith leaders’ messages about HIV and same-sex relationships are associated with acceptance of HIV prevention strategies.

**Methods:**

Multivariable Poisson regression assessed whether attending religious services, faith leaders’ messages about HIV and same-sex relationships, and supportiveness of those messages were associated with HIV testing, as well as knowledge of and willingness to use pre-exposure prophylaxis (PrEP) among 868 Black Americans [45% men; M (SD) = 34 (9) years-old] in the 2016 National Survey on HIV in the Black Community, USA.

**Results:**

Participants who reported attending services monthly and/or hearing faith leaders’ messages that were supportive of same-sex relationships had a significantly higher likelihood of willingness to use PrEP (adjusted Rate Ratio[ARR] = 1.76; 95% confidence interval [CI] = 1.09, 2.48) and aRR = 2.19; 95% CI = 1.35, 3.55, respectively), independent of HIV risk. Homophobia was significantly associated with higher likelihood of being aware of PrEP and testing for HIV testing in the past 12 months.

**Conclusions:**

Faith leaders’ messaging can influence Black Americans’ perceptions and uptake of HIV prevention strategies. Faith institutions and faith leaders should be involved in designing and disseminating HIV prevention strategies.

## Background

HIV infection remains a significant public health problem for Black Americans in the United States (US). Black Americans represent 13% of the US population, yet compared to white people, accounted for 45% of new HIV infections (rate of 44.3 vs 5.3 per 100,000) and 53% of deaths attributed to HIV (rate of 17.4 vs 2.4 per 100,000) in 2015 [1]. Though Black individuals do not engage in higher risk behaviors than people of other races or ethnicities [2], racial disparities persist due to assortative mixing and delays in accessing, testing, treatment, and preventive services. Reducing racial disparities in HIV infection therefore requires addressing testing/screening, access to quality care, and other social and structural determinants [3] not only in groups with high HIV risk, but among the general population [4].

Religion is broadly defined as an organized system of beliefs, rituals, symbols, and lifestyles [5]. Religiousness characterizes the intensity of one’s practices in beliefs and rituals [6]. One recent systematic reviews in 2016 showed that in more than 67% of studies included; at least one aspect of religion was associated with improved health and clinical HIV outcomes among people living with HIV [7]. Regarding prevention, higher HIV testing rates have been noted among those who are more religiously involved [8].

Religion and religiousness are critical social determinants of health for Black Americans. Historically, Black American churches have played central roles in the political and social progress of Black people in the US [9]. According to the most recent (2014) Pew Forum Religious Landscape Survey, 47% of Black Americans attend religious services at least once weekly and 75% say that religion is important in their daily lives [10]. Black American faith leaders play important roles in shaping individual’s attitudes and social norms about HIV, and faith leaders are willing to engage in HIV prevention programs and research [11–13].

Religious service attendance is hypothesized to shape health through mechanisms such as promotion of positive health behaviors [14] including those related to HIV through provision of social support and psychological effects such as boosting self-esteem, coping, and sense of purpose in life [15]. Faith institutions can influence health and behaviors through health ministries, which provide health and social services that can lead to improved access to health information, change in behaviors, and promote better health among individuals in the communities they serve [16].

Faith leaders’ messages are key mechanisms through which religious service attendance shapes HIV related behaviors, especially among Black Americans [17, 18]. Thus faith institutions may be a platform to develop and disseminate HIV prevention messaging and intervention activities [19] to reduce disparities in the HIV/AIDS epidemic especially in the context of new HIV prevention technologies. Pre- Exposure Prophylaxis (PrEP) is a biomedical intervention shown to prevent HIV transmission among at risk individuals by taking one pill once daily (tenofovir/emtricitabine) [20]. PrEP uptake remains low among Black people [21]. One recent study that used medical claims data estimated that 43.7% of black/Africans and 26% whites were eligible for PrEP, but among current users of PrEP who reported race/ethnicity, 13% were black compared to 69% white [22]. Other independent data from CDC shows a bleaker outlook on racial disparities in PrEP. According to the report, only 1% of black people were prescribed PrEP out of 44% black/African Americans who could benefit from PrEP. To date, the social determinants of low PrEP uptake is insufficiently understood.

Given the importance of faith leaders messaging in relation to health behaviors including those that relate to HIV, this study investigated the role of religious service attendance and faith leaders messaging in association with awareness and willingness to use PrEP and HIV testing, among Black Americans. We also assessed individuals’ views about homosexuality because homophobia is prevalent in Black secular and faith communities [23]. We hypothesize that higher frequency of attending religious services and hearing any faith leaders’ messages about HIV or same-sex relationships, especially messages that are supportive, would be associated with higher awareness and willingness to use PrEP and HIV testing, independent of one’s HIV risk and externalized homophobia.

## Methods

### Sample

The National Survey on HIV in the Black Community (NSHBC) included Black Americans between the ages of 18 and 50, who were interviewed between February and April 2016. Participants were recruited primarily through address-based sampling to include households served by cell phones and without landline telephones. Households were provided with internet access and a computer, if needed. Additional details regarding the web-based probability sample recruitment are available [24].

Participants completed a short demographic survey to allow for panel sampling and weighting. Surveys were administered to eligible participants via email. A total of 1969 persons were sampled; 49% (*n* = 970) completed the survey and among those, 89% (*n* = 868) were eligible and completed the study. Post-stratification weights were created so that estimates were representative of adults living in households in the United States according to benchmarks from the latest March 2016 supplement of the Current Population Survey.

### Ethics approval

We obtained written informed consent from all participants prior to their participating in the study. Specifically, participants were shown information about the study along with a consent screen. They had to consent by clicking the written statement “I agree to participate” or else the survey was terminated. Boston Children’s Hospital Institutional Review Board Ethics Committee approved all study protocols including the method of participant consent.

### Measures

#### Religion-related variables

Frequency of attending religious services was assessed with the question, “How often do you attend (or watch TV or online, listen to on the radio) religious service (any denomination, any type of religious service)?” Categories were weekly or greater, monthly, a few times per year, and never. Any faith leaders’ messages about HIV was assessed with the question, “In the past 12 months, did you hear a pastor or any other ministerial staff discuss HIV in sermons or other activities?” Response options were yes, no, or unsure they heard (hereafter unsure). We added a category for those who never attend to retain cases in the analysis, which served as the referent group.

Any faith leaders’ messages about same-sex relationships was assessed with the question, “In the past 12 months, did you hear a pastor or any other ministerial staff discuss same-sex relationships or gay/homosexual people in sermons or other activities?” Response options were similar as the question above and we also derived a new variable following the coding above.

Supportiveness of faith leaders’ messages about HIV was assessed with the question, “Would you say that what was said was supportive or not supportive of people living with HIV?” Response options were supportive, not supportive, unsure. We added a category for those who never attend (reference group) and those who attended but did not respond, to retain cases in multivariable analyses.

Supportiveness of faith leaders’ messages about same-sex relationships was assessed with the question, “Would you say that what was said was positive or negative about homosexuality or same-sex relationships?” Response options were positive, negative, unsure. We used a similar coding strategy as the variable above.

#### HIV prevention variables

PrEP awareness was assessed with the question, “There is a pill (drug/medication) that you can get from your doctor daily to prevent transmission of HIV from an infected (HIV positive) sex partner to an uninfected (HIV negative) partner?” Responses were true, false, don’t know. A new binary variable was derived by collapsing the last two categories (true vs false/don’t know).

PrEP willingness was assessed with the question, “If a pill (drug/medication) that could prevent transmission of HIV from an infected (HIV positive) partner to an uninfected (HIV negative) partner were available, I would take it.” Responses were yes, no, maybe. A binary variable was also derived (yes vs no/maybe) because were most interested in the “yes” response.

Ever tested for HIV was assessed with the question, “Have you ever been tested for HIV?” Responses were yes, no, unsure. A new binary variable was derived by collapsing the last two categories (yes vs else).

Tested for HIV in the past 12 months was assessed with the question, “When was your most recent HIV test?” Responses included within the last 1 year, 1 year to 5 years ago, 6 to 10 years ago, and 11 to 20 years ago. A new binary variable was derived by collapsing the last three categories (within the last 12 months vs else) among the full sample.Covariates.

Covariates included: age (as a continuous variable in years); gender (men, women); marital status (married, divorced/widowed/separated, unmarried); educational attainment (high school, some college, bachelor’s degree or higher); census region (Northeast, Midwest, South, West); time in the USA (less than 10 years, 10 years and more, born in the USA). We included depressive symptoms (derived from the question “During the past month, how often have you been bothered by feeling down, depressed, or hopeless?” Responses, not at all, several days more than half the days, nearly every day). HIV risk classified as meeting criteria for (more than one sexual partner in the last 3 months; or more than one sexual partner and no condom use in the last 3 months; or more than one sexual partner, anal sex and no condom use in the last 3 months; and/or were diagnosed with a sexually transmitted infection (STI) (gonorrhea, chlamydia, herpes, syphilis, trichomoniasis, genital warts, human papilloma virus or HPV) in the 3 months prior to the survey; and/or male-male sexual behavior; and/or transgender (M to F); and/or illicit drug use (e.g., powder or crack cocaine, heroin, or crystal meth] use over the lifetime; or any transactional sexual behavior).

Prejudice, individual or institutional, against lesbians and gay people is defined as homophobia [25]. We assessed homophobia using the Attitudes Toward Gay Men subscale (ATG-R) [25]. We included seven of 10 items from the scale based on cognitive interviews with a convenience sample of 30 self-identified Black individuals ages 18 to 50 in Boston. A 5-point Likert-type assessed responses that ranged from strongly agree to strongly disagree. We derived a composite variable by aggregating the mean of the seven items, which had high internal consistency (i.e., alpha = 0.89) in this sample.

### Statistical analyses

We described the distribution of the sample using survey weighted means and standard deviation for continuous variables and un-weighted n’s and weighted column percent for categorical variables. We then assessed the bivariate associations between the religion, homophobia, and other variables, separately, with each HIV prevention variable. For multivariable analysis, covariates at *p* < 0.20 were considered and all religion and homophobia variables were included regardless of statistical significance. We used Log-Poisson regression to approximate the prevalence ratio (PR) for binary variables. Statistical significance was evaluated through 95% confidence intervals. We conducted multivariable models for each HIV prevention variable by assessing each religion variable separately. Next, given that Black women are more religiously involved than men, in secondary analyses, we examined potential effect modification by gender with the religion variables on each HIV prevention outcome. However, we did not find a significant association, so gender was included as main effect variable in the models where significant in bivariate analyses. Next, in secondary analyses, we examined whether the association between one’s homophobia was modified by religious attendance and faith leader’s messages. No effect modification was present with any of the HIV prevention variables, so homophobia was included as a main effect variable.

## Results

### Descriptive statistics and bivariate tests

Table 1 describes the characteristics of the sample and the bivariate associations. The mean age was 34 years (SD = 9), 45% of the sample were men, 20% had a bachelor’s degree or higher and 54% resided in the Southern region. Seventy three percent of participants attended religious services, 8% heard a faith leader’s message about HIV, and 30% a message about same-sex relationships. The mean homophobia score was 2.88 (SD = 1.1) that ranged between 1 for low and 7 for high.

**Table 1:** Descriptive and univariate association between religion variables, sociodemographic covariates and HIV prevention variables in the National Survey on HIV in the Black American Community (NSHBC)

Religion variables	M (SD) or N (col %)	PrEP Awareness (1 = aware)	PrEP Willingness (1 = willing)	Ever Tested for HIV (1 = yes)	Recent HIV Testing^d^(1 = yes)
		PR (95% CI)	PR (95% CI)	PR (95% CI)	PR (95% CI)
Frequency of attending religious services					
Never attend	227 (0.27)	1	1	1	1
A few times a year	20 (0.23)	1.39 (0.78, 2.46)	1.55 (1.08, 2.46)	1.28 (1.09, 1.51)	0.99 (0.69, 1.41)
Monthly	87 (0.11)	1.64 (0.84, 3.20)	1.55 (0.96, 2.49)	1.25 (1.02, 1.52)	0.98 (0.63, 1.53)
Weekly and greater	348 (0.39)	1.30 (0.78, 2.16)	0.91 (0.63, 1.32)	1.13 (0.96, 1.34)	0.81 (0.59, 1.12)
Any faith leaders’ message about HIV					
Never attend	227(0.27)	1	1	1	1
Yes, heard	62 (0.08)	1.81 (0.92, 3.55)	1.57 (0.94, 2.64)	1.28 (1.03, 1.59)	0.81 (0.45, 1.45)
No	514 (0.58)	1.36 (0.85, 2.16)	1.21 (0.87, 1.69)	1.16 (0.99, 1.35)	0.81 (0.45, 1.45)
Unsure	58 (0.07)	1.11 (0.35, 3.54)	0.86 (0.40, 1.87)	1.36 (1.12, 1.65)	0.91 (0.68, 1.22)
Any faith leaders’ messages about same-sex relationships					
Never attend	227 (0.27)	1	1	1	1
Yes, heard	255 (0.30)	1.63 (0.98, 2.70)	1.19 (0.82, 1.74)	1.27 (1.08, 1.49)	0.73 (0.52, 1.03)
No	333 (0.37)	1.05 (0.61, 1.77)	1.29 (0.91, 1.84)	1.13 (0.95, 1.33)	0.85 (0.46, 1.61)
Unsure	46 (0.06)	2.05 (0.90, 4.67)	0.76 (0.35, 1.62)	1.23 (0.97, 1.55)	0.95 (0.70, 1.28)
Supportiveness of faith leaders’ messages about HIV					
Never attend	227 (0.27)	1	1	1	1
Attend, no response^c^	579 (0.65)	1.31 (0.82, 2.09)	1.16 (0.84, 1.61)	1.19 (1.02, 1.38)	0.85 (0.46, 1.61)
Yes, supportive	41 (0.05)	1.95 (0.90, 4.25)	1.70 (0.96, 3.00)	1.14 (0.85, 1.54)	0.98 (0.51, 1.87)
No	5 (0.01)	Omitted	Omitted	Omitted	Omitted
Unsure	16 (0.02)	1.04 (0.29, 3.68)	1.33 (0.43, 4.17)	1.54 (1.30, 1.82)	0.92 (0.69, 1.23)
Supportiveness of faith leaders’ messages about same-sex relationships					
Never attend	227 (0.26)	1	1	1	1
Attend, no response^c^	387 (0.43)	1.20 (0.72, 2.00)	1.20 (0.85 1.70)	1.15 (0.98, 1.35)	0.85 (0.46, 1.61)
Yes, supportive	38 (0.04)	1.43 (0.62, 3.27)	2.17 (1.33, 3.53)	1.29 (1.03, 1.62)	0.85 (0.46, 1.61)
No	128 (0.15)	1.97 (1.10, 3.51)	1.20 (0.75, 1.90)	1.21 (0.99, 1.46)	0.74 (0.51, 1.07)
Unsure	88 (0.10)	1.12 (0.52, 2.40)	0.79 (0.44, 1.42)	1.35 (1.13, 1.61)	1.02 (0.76, 1.39)
ocio demographics					
Age	33.63 (9)	0.97 (0.95, 0.99)	1.00 (0.98, 1.01)	1.01 (1.00, 1.12)	0.98 (0.97, 1.00)
Gender					
Men	346 (0.45)	1	1	1	1
Women	522 (0.55)	0.85 (0.56, 1.28)	1.05 (0.78, 1.40)	1.18 (1.05, 1.33)	1.11 (0.84, 1.46)
Marital Status					
Married	243 (0.30)	1	1	1	1
Divorce/Widowed/Separated	86 (0.09)	1.26 (0.55, 2.87)	1.72 (0.96, 3.09)	1.13 (0.99, 1.30)	1.59 (0.99, 2.55)
Unmarried	539 (0.61)	1.45 (0.91, 2.30)	1.75 (1.20, 2.54)	0.90 (0.79, 1.02)	1.79 (1.30, 2.46)
Educational attainment					
Less than high school	62 (0.11)	0.75 (0.35, 1.57)	2.10 (1.34, 3.28)	0.94 (0.77, 1.14)	0.77 (0.44, 1.33)
High school	179 (0.33)	0.59 (0.34, 1.02)	1.11 (0.73, 1.69)	0.78 (0.66, 0.92)	1.02 (0.72, 1.44)
Some college	353 (0.36)	0.67 (0.43, 1.03)	1.55 (1.12, 2.16)	0.98 (0.88, 1.08)	0.96 (0.74, 1.25)
Bachelor’s degree or higher	274 (0.20)	1	1	1	1
Time in the USA					
Less than 10 years	20 (0.02)	1.34 (0.50, 3.57)	0.53 (0.14, 2.03)	1.32 (1.21, 1.45)	0.71 (0.31, 1.61)
10 years and more	81 (0.09)	1.33 (0.77, 2.30)	0.88 (0.53, 1.45)	0.79 (0.62, 1.00)	1.04 (0.68, 1.60)
Born in the USA	767 (0.89)	1	1	1	1
Census Region					
Northeast	152 (0.18)	1.21 (0.70, 2.07)	0.70 (0.45, 1.08)	0.95 (0.08, 1.12)	1.10 (0.78, 1.54)
Midwest	175 (0.17)	0.84 (0.49, 1.44)	1.03 (0.72, 1.49)	0.91 (0.77, 1.07)	0.85 (0.60, 1.22)
South	439 (0.54)	1	1	1	1
West	102 (0.11)	0.52 (0.24, 1.10)	1.28 (0.84, 1.93)	1.00 (0.85, 1.18)	1.13 (0.75, 1.72)
Other variables					
Depressive symptoms [1 to 4]	1.58 (0.80)	1.08 (0.86, 1.36)	1.21 (1.06, 1.42)	1.02 (0.95, 1.10)	1.05 (0.91, 1.22)
HIV risk^a^					
Yes	211 (0.23)	2.18 (1.45, 3.25)	1.18 (1.39, 2.41)	1.32 (1.20, 1.45)	1.12 (0.84, 1.46)
No	657 (0.77)	1	1	1	1
Homophobia^b^ [1 to 5]	2.88 (1.10)	1.34 (1.10, 1.63)	1.13 (0.99, 1.29)	0.99 (0.94, 1.05)	1.18 (1.04, 1.33)
IV-prevention variables					
PrEP Awareness					
Yes, aware	136 (0.15)				
PrEP Willingness					
Yes, willing to use	234 (0.27)				
Ever tested for HIV					
Yes	627 (0.71)				
Recent HIV testing^d^					
Yes	253 (0.28)				

Notes. *M* mean, *SD* standard deviation, *N* unweighted sample, col% based on weighted data. *PR* Prevalence Ratio, *CI* Confidence Interval. a=HIV Risk variable is a derived variable corresponding to >1 sexual partner in the past 3 months or anal sex & greater than 1 partner or STD in the past 3 months or lifetime illicit drug use or > 1 sexual partner in the past 3 months, and no condom use, or any transactional sex. b=homophobia is the mean of a seven-item scale measuring ones’ view of same sex couples and homosexuality. c=a few people attended but had no responses to the question. d=Tested for HIV in the past 12 months among all participants.

PrEP=Pre-exposure Prophylaxis. Omitted in the regression because of small cell count.

In bivariate analysis, compared to non-attenders, those who attended religious services a few times a year or more were more willing to use PrEP and to have been tested for HIV. Compared to non-attenders, those who reported hearing any faith leaders’ message about HIV or same-sex relationships were more likely to have been ever tested for HIV. Compared to non-attenders, those who reported hearing supportive messages about same-sex relationships were more likely willing to use PrEP.

### Multivariable regressions

Tables 2, 3 and 4 describes results from the multivariable associations of the religion variables in association with the HIV prevention variables (i.e., PrEP awareness and willingness, and HIV testing) independent one’s HIV risk status, homophobia, and covariates. Religious service attendance.

**Table 2:** Multivariable relationship between attending religious services, and individual homophobia, in association with each HIV prevention variable in the National Survey on HIV in the Black American Community (NSHBC)

Religion variable	PrEP Awareness (1 = aware)	PrEP Willingness (1 = willing)	Ever Tested for HIV (1 = yes)	Recent HIV testing^a^(1 = yes)
	aPR (95% CI)	aPR (95% CI)	aPR (95% CI)	aPR (95% CI)
Frequency of attending religious services				
Never attend	1	1	1	1
A few times a year	1.44 (0.82, 2.51)	1.55 (1.07, 2.22)	1.16 (0.99, 1.36)	1.29 (0.88, 1.89)
Monthly	2.01 (1.05, 3.86)	1.76 (1.09, 2.84)	1.18 (0.96, 1.44)	1.40 (0.88, 2.20)
Weekly and greater	1.98 (1.22, 3.21)	1.11 (0.76, 1.63)	1.04 (0.89, 1.22)	1.12 (0.77, 1.63)
Homophobia	1.35 (1.14, 1.60)	1.08 (0.94, 1.23)	1.00 (0.94, 1.04)	1.18 (1.04, 1.34)
	Adjusted for age and HIV risk	Adjusted for education, marital status, depression and HIV risk	Adjusted for age, gender, education, time in the US, and HIV risk	Adjusted for age, marital status, and HIV risk forced into the model

Notes. *aPR* Adjusted Prevalence Ratio, *CI* Confidence Interval

Covariates in the adjusted models are based on those significant in the bivariate models at *p* < 0.20. Estimates were unaltered in a model without HIV risk, which was not significant in the bivariate model

Homophobia is the mean of a seven-item scale measuring ones’ view of same sex couples and homosexuality

a=Tested for HIV in the past 12 months among all persons

**Table 3:** Multivariable relationship between any faith leader’s message about HIV, supportiveness of those messages, and individual homophobia, in association with each HIV prevention variable in the National Survey on HIV in the Black American Community (NSHBC)

Religion variable	PrEP Awareness (1 = aware)	PrEP Willingness (1 = willing)	Ever Tested for HIV (1 = yes)	Recent HIV testing^a^(1 = yes)
	aPR (95% CI)	aPR (95% CI)	aPR (95% CI)	aPR (95% CI)
Any faith leader’s message about HIV				
Never attend	1	1	1	1
Yes, heard	2.06 (1.03, 4.13)	1.62 (0.96, 2.85)	1.15 (0.93, 1.43)	1.12 (0.60, 2.10)
No	1.71 (1.09, 2.67)	1.41 (1.02, 1.97)	1.08 (0.93, 1.26)	1.19 (0.85, 1.66)
Unsure	1.60 (0.50, 5.16)	0.92 (0.46, 1.84)	1.27 (1.05, 1.54)	1.96 (1.14, 3.35)
Homophobia	1.31 (1.09, 1.57)	1.12 (0.98, 1.27)	1.00 (0.96, 1.06)	1.16 (1.03, 1.31)
	Adjusted for age and HIV risk	Adjusted for education, marital status, depression and HIV risk	Adjusted for age, gender, education, time in the US, and HIV risk	Adjusted for age, marital status, and HIV risk forced into the model
^b^Supportiveness of faith leader’s message about HIV				
Never attend services	1	1	1	1
Attend, no response	1.68 (1.07, 2.62)	1.35 (0.97, 1.87)	1.11 (0.96, 1.29)	1.25 (0.90, 1.74)
Yes, supportive	2.09 (0.94, 4.63)	1.76 (0.97, 3.18)	1.04 (0.79, 1.36)	1.11 (0.53, 2.33)
No	Omitted	Omitted	Omitted	Omitted
Unsure	1.45 (0.35, 5.93)	1.76 (0.61, 5.07)	1.43 (1.15, 1.79)	0.70 (0.21, 2.30)
Homophobia	1.40 (1.09, 1.79)	1.13 (0.99, 1.28)	1.00 (0.94, 1.04)	1.16 (1.01, 1.33)
	Adjusted for age and HIV risk	Adjusted for education, marital status, depression and HIV risk	Adjusted for age, gender, education, time in the US, and HIV risk	Adjusted for age, marital status, and *HIV risk forced into the model

Notes. *aPR* Adjusted Prevalence Ratio, *CI* Confidence Interval

Covariates in the adjusted models are based on those significant in the bivariate models at *p* < 0.20

*Estimates were unaltered in a model without HIV risk, which was not significant in the bivariate model

Homophobia is the mean of a seven-item scale measuring ones’ view of same sex couples and homosexuality

a=Tested for HIV in the past 12 months among all participants. Omitted in the regression because of small cell count

b=fit in a separate regression model but presented in the same table

**Table 4:** Multivariable relationship between any faith leader’s message about same-sex relationships, supportiveness of those messages, and individual homophobia, in association with each HIV prevention variable in the National Survey on HIV in the Black American Community (NSHBC)

Religion variable	PrEP Awareness (1 = aware)	PrEP Willingness (1 = willing)	Ever Tested for HIV (1 = yes)	Recent HIV testing^a^(1 = yes)
	aPR (95% CI)	aPR (95% CI)	aPR (95% CI)	aPR (95% CI)
Any faith leader’s message about same-sex relationships				
Never attend	1	1	1	1
Yes, heard	2.21 (1.30, 3.71)	1.40 (0.94, 2.07)	1.164 (0.97, 1.34)	1.15 (0.77, 1.70)
No	1.29 (0.78, 2.14)	1.48 (1.05, 2.10)	1.07 (0.92, 1.25)	1.17 (0.82, 1.66)
Unsure	2.79 (1.18, 6.57)	0.78 (0.38, 1.62)	1.20 (0.93, 1.55)	2.17 (1.29, 3.67)
Homophobia	1.35 (1.12, 1.62)	1.12 (0.98, 1.27)	1.00 (0.95, 1.07)	1.21 (1.06, 1.38)
	Adjusted for age and HIV risk	Adjusted for education, marital status, depression and HIV risk	Adjusted for age, gender, education, time in the US, and HIV risk	Adjusted for age, marital status, and HIV risk forced into the model
^b^Supportiveness of faith leader’s message about same-sex relationships				
Never attend services	1	1	1	1
Attend, no response	1.48 (0.91, 2.38)	1.37 (0.97, 1.93)	1.10 (0.94, 1.28)	1.31 (0.93, 1.85)
Yes, supportive	1.73 0.80, 3.70)	2.19 (1.35, 3.55)	1.05 (0.84, 1.31)	1.20 (0.64, 2.25)
No	2.56 (1.42, 4.62)	1.38 (0.85, 2.24)	1.11 (0.92, 1.34)	1.05 (0.64, 1.72)
Unsure	1.72 (0.78, 3.79)	1.02 (0.55, 1.87)	1.22 (1.03, 1.47)	1.17 (0.69, 1.97)
Homophobia	1.34 (1.11, 1.61)	1.12 (0.99, 1.28)	1.00 (0.94, 1.06)	1.15 (1.00, 1.33)
	Adjusted for age and HIV risk	Adjusted for education, marital status, depression and HIV risk	Adjusted for age, gender, education, time in the US, and HIV risk	Adjusted for age, marital status, and *HIV risk forced into the model

Notes. *aPR* Adjusted Prevalence Ratio, *CI* Confidence Interval

Covariates in the adjusted models are based on those significant in the bivariate models at *p* < 0.20

*Estimates were unaltered in a model without HIV risk, which was not significant in the bivariate model

Homophobia is the mean of a seven-item scale measuring ones’ view of same sex couples and homosexuality

a = Tested for HIV in the past 12 months among all participants

b = fit in a separate regression model but presented in the same table

Compared to non-attenders, those who attended services a few times a year (adjusted Prevalence Ratio (aPR) = 1.55, 95% Confidence Interval (CI) = 1.07, 2.22) and monthly (aPR = 1.76, 95%CI = 1.09, 2.84) were more likely to be willing to use PrEP. Service attendance was not statistically associated with any of the other HIV prevention variables (Table 2).

#### Any faith leaders’ messages about HIV, and supportiveness of those messages

Compared to non-attenders, persons who reported hearing faith leaders’ messages about HIV (aPR = 2.06, 95%CI = 1.03, 4.13) as well as those who said no (aPR = 1.17, 95%CI = 1.09, 2.67) had higher awareness of PrEP (aPR = 3.57, 95%CI = 2.15, 5.93), but those who attended church, but did not hear a message were more likely to be willing to use PrEP (aPR = 1.41, 95%CI = 1.02, 1.97). Compared to non-attenders, those who said unsure were statistically more likely to have ever been tested for HIV as well as in the past 12 months (Table 3). Compared to non-attenders and persons who attended but did not respond were more likely to be aware of PrEP (aPR = 1.68, 95%CI = 1.07, 2.62) (Table 3).

#### Any faith leaders’ messages about same-sex relationships, and supportiveness of those messages

Compared to non-attenders, those who reported hearing about same-sex relationships (aPR = 2.21, 95%CI = 1.30, 3.71) as well as those who said unsure (aPR = 2.79, 95%CI = 1.18, 6.57) were more likely to be aware of PrEP (Table 4), however, only those who said no to hearing a message was more likely willing to use PrEP (aPR = 1.48, 95%CI = 1.05, 2.10), and those who said unsure about hearing a message more likely to have tested for HIV in the past 12 months (aPR = 2.17, 95%CI = 1.29, 3.67) (Table 4). Compared to non-attenders, those who reported the message was not supportive were statistically more likely to be aware of PrEP (aPR = 2.56, 95%CI = 1.42, 4.62), those who said the message was supportive were statistically more likely to be willing to use PrEP (aPR = 2.19, 95%CI = 1.35, 3.55). Those who said unsure about hearing a message more likely to have ever tested for HIV (aPR = 1.22, 95%CI = 1.03, 1.47) (Table 4).

#### Homophobia

Higher levels of homophobia were significantly associated with higher likelihood of being aware of PrEP and higher likelihood of being tested for HIV in the past 12 months, independent of each religion variable and one’s HIV risk, and covariates (see Tables 2, 3 and 4).

## Discussion

### Overview

Religion plays a prominent role in the lives of Black Americans and has been typically been associated with protective health behaviors. HIV burden is high in the Black American population compared to other racial/ethnic groups and thus we sought to investigate the roles of religious service attendance and faith leaders’ messages on HIV and same-sex relationships in association with knowledge and willingness to use HIV prevention. Our findings are timely in the context of HIV prevention because the Food and Drug Administration approved tenofovir-emtricitabine for PrEP—a biomedical HIV prevention technology in 2012, yet uptake of PrEP remains woefully slow, particularly among Black people [21].

### Review of findings with potential explanations and implications

Compared to those who do not attend religious services, people who attended religious services a few times a year or monthly had higher likelihoods of being willing to use PrEP. The positive association between attendance and being willing to use PrEP could be related to the presence of HIV or other health ministries within some religious or faith institutions that provide knowledge and education about HIV-related health services [23]. Therefore, regular attendance could increase one’s likelihood of receiving educational information about the benefits of PrEP.

Beyond examining the role of attending religious services, we advance an understanding of the religion-health connection by quantitatively examining the role of faith leaders messaging about HIV and same-sex relationships. In one study among African American churches in South Carolina, HIV messaging occurred in 10.6% of all messages related to chronic and infectious diseases [26]. In our study, eight-percent of respondents reported hearing messages about HIV within the service. Those who reporting hearing the messages were more likely to be aware of PrEP, but not statistically more likely to be willing to use PrEP. Those who were unsure whether they heard a message about HIV were more likely to test for HIV. We speculate that external influences beyond the services in the church or the general faith community, perhaps HIV prevalence in one’s community, could have influenced one’s likelihood to test for HIV despite not hearing a specific message. In addition, the degree of supportiveness of the HIV-related message was unrelated to one’s willingness to use PrEP. Future qualitative work is necessary to understand this dynamic and to generate more nuanced hypotheses.

Thirty percent of NSHBC respondents reported that they heard messages about same-sex relationships. In a 2016 PEW national study, 39% of respondents who attended religious services reported hearing clergy discuss homosexuality [27]. Participants in our study who reported hearing those messages or were neutral had higher likelihoods of being aware of PrEP. We speculate that those who reported that they were unsure about the messages could have likely been exposed to PrEP from friends or family with a greater intensity or frequency than in the messages from the religious setting.

Those who reported hearing positive messages about same-sex relationships had significantly higher likelihoods of being willing to use PrEP independent of their own homophobia and HIV risk. In a study designed to determine the efficacy of a church based HIV prevention intervention (including messages from clergy), Berkley-Patton et al. found a twofold greater HIV testing rates among parishioners in faith institutions that received the intervention compared to those in the control group with no or limited HIV messaging [19]. Similar interventions can potentially be adapted to improve PrEP uptake in this setting. We recommend future qualitative research to understand the context and content of what a supportive message about same-sex relationship entails and to delineate pathways to behavior change around PrEP. Our finding is also important for diffusion of attitudes within a social network. For instance, Wingood et al. found that Black American women were more willing to use PrEP if their female friends would use it [28], which suggests trusted peers are important conduits to promote adopting this new HIV prevention technology.

### Study limitations

Several limitations of this study should be noted. The NSHBC was a cross-sectional survey, so we could not assess temporal associations or causality between the religion and the HIV prevention variables. The survey sample was nationally representative but did not specifically recruit individuals at highest risk of HIV infection, nor those who were homeless, transiently housed or those in institutionalized populations.

Related to the topic of high-risk, we did not stratify the statistical analysis to obtain estimates solely among individuals deemed as HIV risk status (e.g., MSM)—who are the Centers for Disease and Prevention (CDC)‘s priority target for PrEP. To reduce confounding by HIV risk status, we statistically adjusted for this variable based on our rationale that we wanted to focus on the general population.

We did not examine a range of potential effect modifiers in this analysis. While we examined potential effect modification by gender, it is possible that age, socioeconomic status, or religious denomination may moderate the associations between the religion variables and the HIV prevention variables. For instance, religious attendance among Black Americans are known to increase with age, and those with higher education and income are more mobile and empowered and may be less likely to worship in faith communities where messages are not aligned with equality around same-sex relationships. The Black church is also not a monolith but comprised of diverse ethnicities, cultures, denominations, and beliefs [10]. We plan to examine effect modification in future analyses where we can further develop potential hypotheses to test.

There is potential for social desirability biases because some respondents might want to convey high frequency of attending services. We did not have an objective way to measure frequency of attendance, which is a challenge in most religion and health studies today. Nevertheless, our self-reported prevalence of attending services once a week or more was 39%, which is similar to 38% from The National Survey of American Life (NSAL)—one of the largest probability surveys on Black Americans in the US [29]. The high concordance between our prevalence estimate and NSAL’s exhibits high concordance and external validity. In addition, in our study, 38% self-reported testing for HIV in the past 12 months which is reasonably close to 32% in the Behavioral Risk Factor Surveillance System—one well-respected large national survey [30]. This close correlation of prevalence estimates increases our confidence in the study results. Relatedly, the 12-month timeframe of our study could possibly induce recall bias, which if occurred would most likely underestimate our measures of associations.

While the study contained several innovative religion variables (i.e., questions about faith leaders’ messages about HIV and same-sex relationships), the study did not contain other multidimensional measures such as spirituality, which is also likely to have an independent impact on HIV prevention variables [31].

## Conclusions

There are approximately 46 million Black Americans in the US and 47% report attending religious services weekly, which means there are potentially 21.6 million people that can influence behavior change. Given that faith leaders are willing to engage in HIV prevention programs and research [11–13], Faith institutions and faith leaders should be involved in designing and disseminating HIV prevention strategies for Black Americans.

## Abbreviations

ATG-R: Attitudes Toward Gay Men
CI: Confidence interval
NSAL: National Survey of American Life
NSHBC: National Survey on HIV in the Black Community
PR: Prevalence ratio
PrEP: Pre-exposure prophylaxis
STI: sexually transmitted infection
US: United States

## References

[1] Centers for Disease Control and Prevention: HIV surveillance report, volume 27: diagnosis of HIV infection in the United States and dependent areas*.* Retrieved December 19, 2016 from https://www.cdc.gov/hiv/pdf/library/reports/surveillance/cdc-hiv-surveillance-report-2015-vol-27.pdf

[2] Hallfors DD, Iritani BJ, Miller WC, Bauer DJ (2007). Sexual and drug behavior patterns and HIV and STD racial disparities: the need for new directions. Am J Public Health.

[3] Aral SO, Adimora AA, Fenton KA (2008). Understanding and responding to disparities in HIV and other sexually transmitted infections in African Americans. Lancet.

[4] Coates TJ, Richter L, Caceres C (2008). Behavioural strategies to reduce HIV transmission: how to make them work better. Lancet.

[5] Geertz C, Lambek M (2008). Religionas a cultural system. A reader in the anthropology of religion.

[6] Zinnbauer BJ, Pargament KI, Cole B (1997). Religion and spirituality: unfuzzying the fuzzy. J Sci Study Relig.

[7] Doolittle BR, Justice AC, Fiellin DA. Religion, spirituality, and HIV clinical outcomes: a systematic review of the literature. AIDS Behav. 2016;22(6):1792–801. 10.1007/s10461-016-1651-z 28004218

[8] Ludema C, Doherty IA, White BL (2015). Religiosity, spirituality, and HIV risk behaviors among African American women from four rural counties in the southeastern US. J Health Care Poor Underserved.

[9] Lincoln EC, Mamiya LH (1990). The black church in the African American experience.

[10] Pew Forum on Religion and Public Life (2014). Religious Landscape Study: racial and ethnic composition.

[11] Foster PP, Cooper K, Parton JM, Meeks JO (2011). Assessment of HIV/AIDS prevention of rural African American Baptist leaders: implications for effective partnerships for capacity building in American communities. J Natl Med Assoc.

[12] Nunn A, Cornwall A, Thomas G (2013). What’s God got to do with it? engaging African-American faith-based institutions in HIV prevention. Glob Public Health.

[13] Berkley-Patton J, Thompson CB, Martinez DA (2013). Examining church capacity to develop and disseminate a religiously appropriate HIV tool kit with African American churches. J Urban Health.

[14] Strawbridge WJ, Shema SJ, Cohen RD, Kaplan GA (2001). Religious attendance increases survival by improving and maintaining good health behaviors, mental health, and social relationships. Ann Behav Med.

[15] Ellison CG, Levin JS (1998). The religion-health connection: evidence, theory, and future directions. Health Educ Behav.

[16] DeHaven MJ, Hunter IB, Wilder L, Walton JW, Berry J (2004). Health programs in faith-based organizations: are they effective?. Am J Public Health.

[17] Nunn A, Cornwall A, Chute N (2012). Keeping the faith: African American faith leaders’ perspectives and recommendations for reducing racial disparities in HIV/AIDS infection. PLoS One.

[18] Adaora AA, Goldmon MV, Coyne-Beasley T, Ramirez CB, Thompson GA, Ellis D, Stevenson JL, Williams JM, Howard DL, Godley PA. Black Pastors’ Views on preaching about sex: barriers, facilitators, and opportunities for HIV prevention messaging. Ethn Health. 2017. 10.1080/13557858.2017.1346180. 10.1080/13557858.2017.1346180 28670980

[19] Berkley-Patton J, Thompson CB, Moore E (2016). An HIV testing intervention in African American churches: plot study findings. Ann Behav Med.

[20] Centers for Disease Control and Prevention: *Pre-Exposure Prophylaxis (PrEP).* Retrieved September 3, 2017 from http://www.webcitation.org/6tDRkl99I

[21] Mera R, McCallister S, Mayer G, Magnuson D, Rawlings K (2016). Truvada (TVD) for HIV pre-exposure prophylaxis (PrEP) utilization in the United States (2013–2015).

[22] Huang Y, Zhu W, Smith D, Harris N, Hoover K (2018). HIV preexposure prophylaxis, by race and ethnicity--United States, 2014-2016. MMWR Morb Mortal Wkly Rep.

[23] Eke AN, Wilkes AL, Gaiter J, McCree DH, Jones K, O'Leary A (2010). Organized religion and the fight against HIV/AIDS in the black community: the role of the black church. African Americans and HIV/AIDS: understanding and addressing the epidemic.

[24] (GFK) GFK: *Knowledge panel design summary.* Retrieved August 2, 2017 from http://www.webcitation.org/query?url=http%3A%2F%2Fwww.knowledgenetworks.com%2Fganp%2Fdocs%2FKnowledgePanel%28R%29-Design-Summary.pdf&date=2017-08-02

[25] Herek GM (1988). Heterosexuals’ attitudes toward lesbians and gay men: correlates and gender differences. J Sex Res.

[26] Harmon B, Chock M, Brantley E, Wirth M, Hébert J (2016). Disease messaging in churches: implications for health in African-American communities. J Relig Health.

[27] Pew Research Center: *Many Americans hear politics from the pulpit.* Retrieved November 1, 2016 from http://www.webcitation.org/74dWChqXI/.

[28] Wingood GM, Dunkle K, Camp C (2013). Racial differences and correlates of potential adoption of pre-exposure prophylaxis (PrEP): results of a national survey. J Acquir Immune Defic Synd.

[29] Taylor RJ, Chatters LM, Joe S (2011). Religious involvement and suicidal behavior among African Americans and black Caribbeans. J Nerv Ment Dis.

[30] Gaines TL, Caldwell JT, Ford CL, Mulatu MS, Godette DC (2016). Relationship between a Centers for Disease Control and Prevention expanded HIV testing initiative and past-year testing by race/ethnicity: a multilevel analysis of the behavioral risk factor surveillance system. AIDS Care.

[31] Buck AC, Williams DR, Musick MA, Sternthal MJ (2009). An examination of the relationship between multiple dimensions of religiosity, blood pressure, and hypertension. Soc Sci Med.

